# Genomic Analysis of Antibiotics Resistance in Pathogens

**DOI:** 10.3390/antibiotics11081013

**Published:** 2022-07-28

**Authors:** Teresa Nogueira

**Affiliations:** 1 INIAV—National Institute for Agrarian and Veterinary Research, 2780-157 Oeiras, Portugal; teresa.nogueira@iniav.pt; 2 cE3c—Center for Ecology, Evolution and Environmental Change & CHANGE—Global Change and Sustainability Institute, Faculdade de Ciências, Universidade de Lisboa, 1749-016 Lisbon, Portugal

The emergence of antibiotic-resistant pathogens currently represents a serious threat to public health and the economy worldwide. Due to antibiotic treatments in human and veterinary medicine, pathogenic bacteria are most often exposed to unnatural doses of antibiotics and their selective effect.

Back in 1945, in his Nobel prize lecture, Alexander Fleming said: “The time may come when penicillin can be bought by anyone in the shops. Then there is the danger that the ignorant man may easily underdose himself and by exposing his microbes to non-lethal quantities of the drug make them resistant. Here is a hypothetical illustration. Mr. X. has a sore throat. He buys some penicillin and gives himself, not enough to kill the streptococci but enough to educate them to resist penicillin. He then infects his wife. Mrs. X gets pneumonia and is treated with penicillin. As the streptococci are now resistant to penicillin the treatment fails. Mrs. X dies. Who is primarily responsible for Mrs. X’s death? Why Mr. X whose negligent use of penicillin changed the nature of the microbe. Moral: If you use penicillin, use enough” [1].

In fact, after the discovery of penicillin, antibiotics were introduced into clinical practice in humans, and later in veterinary health and agriculture as growth promoters. As a consequence, antibiotics became an environmental contaminant.

Fleming predicted what we know today to be the genomic dynamics of the acquisition of antibiotic resistance and its epidemic nature, eight years before Francis Crick and James Watson determined the structure of the DNA molecule. He also predicted that the widespread, unsupervised use of antibiotics could become a health problem, as he was already aware that antibiotic resistance could be triggered by exposure to sublethal doses of antibiotics.

In bacteria, antibiotic resistance can be encoded on chromosomes, plasmids, or other mobile genetic elements. It can also result from mutations that lead to changes in the affinity of antibiotics for their targets or in the ability of antibiotics to act on bacterial growth or death. It is essential to understand the evolutionary dynamics and mobilization of genes encoding antibiotic resistance in the human, animal, plant, and environmental microbiomes but also the transmission of resistant bacteria between individuals and between humans, animals, and the environment in a One Health approach. These studies can be developed using genomic and metagenomic approaches and bioinformatics analyses. This Special Issue addresses the horizontal transfer of antibiotic resistance genes and their spread, epidemiology, and association with bacterial virulence between bacterial genotypes and their phenotypes.

Although antibiotics are global contaminants, environmental bacteria, commensals, and human and animal pathogens are not expected to be exposed to the same types of antibiotics. Pathogenic bacteria will more often be exposed to therapeutic doses of the antibiotics of medical interest. Darmancier et al. [2] performed a comprehensive bioinformatic study of 16,632 complete bacterial reference genome sequences to discover whether there would be a relationship between bacterial virulence and antibiotic resistance. They found evidence that some categories of virulence and antibiotic resistance genes could be co-selected and that mobile genetic elements, such as integrative and conjugative elements, could play an important role in their co-mobilization. The fact that human pathogens whose therapy requires specific antibiotics have become resistant has emerged as a major obstacle to the treatment of diseases that threaten humans.

This collection also gathers papers on antibiotic resistance of some of the major emerging infections pathogens such as *Mycobacteroides abscessus* [3], *Klebsiella pneumoniae* [4,5], *Salmonella* sp. [6,7], *Francisella tularensis* [8], *Acinetobacter baumannii* [9,10], or *Pseudomonas aeruginosa* [11] and other human pathogens such as *Neisseria gonorrhoea* [12], *Staphylococcus aureus* [13], or *Escherichia coli* [14]. Most of them are high-priority pathogens [15], according to the National Institute of Allergy and Infectious Diseases, and are globally and medically important pathogens.

Ng and Ngeow have published a review on the Tigecycline (a third-generation tetracycline) resistance in the Actinobacteria *Mycobacteroides abscessus* a clinically important human pathogen known to harbor a multidrug-resistance phenotype [3].

*Staphylococcus aureus* is one of the main etiological agents of skin and wound infections of nosocomial origin, belonging to the phylum Firmicutes. It has been classified as a globally and medically important human and foodborne pathogen [16], and methicillin resistance has been a major challenge to treatment and patient science. Ullah et al. have performed a comparative genomic analysis of a highly virulent methicillin-resistant *S. aureus* (MRSA) of a Pakistan clone [13].

*Neisseria gonorrhoeae* is a Beta Proteobacteria that is the etiological agent of gonorrhea, a sexually transmitted disease, as well as an eye infection transmitted, for example, during vaginal delivery, which can lead to blindness when left untreated. Gonzalez et al. conducted a study in which they allowed two reference strains of *N. gonorrhoeae* to evolve and then followed the emergence of ciprofloxacin resistance by mutation. They then performed genomic analyses of several of the clones to conclude that there are strain-specific differences in the emergence of ciprofloxacin resistance [12].

The Gamma Proteobacteria phylum encloses many human pathogens. This collection includes a review by Rodrigues and his colleagues who have characterized the resistome of *Acinetobacter baumannii* and described its genome as very plastic and open [10]. Thadtapong et al. have highlighted the epidemic potential of colistin and carbapenem-resistant *A. baumannii* clone carrying a conjugative system and a potential virulence system [9].

Another Gamma Proteobacteria is *Pseudomonas aeruginosa*, which is an opportunistic human pathogen that is often difficult to treat with antibiotics due to its ability to form biofilms but also due to the expression of efflux pumps. Ahmed et al. have studied the impact of titanium dioxide nanoparticles on the quorum-sensing genes controlling biofilm production and efflux pump gene expression [11].

Souder et al. have identified a novel role for two genes *dipA* and *pilD* in *Francisella tularensis* (the etiologic agent of Tularaemia) susceptibility to resazurin [8].

The phylum Gamma-proteobacteria also includes the most common etiologic agents of human gastroenteritis: *Salmonella* sp. and *Escherichia coli*. Hernández-Díaz and colleagues performed a comparative genomic analysis of Typhimurium serotypes of *S. enterica* that led to the identification of 44 genes, 34 plasmids, and 5 point mutations associated with antibiotic resistance, distributed across 220 genomes of ST213 strains [6]. Vázquez et al., on the other hand, performed a genomic analysis of *S. enterica* serotype Infantis to identify *pESI*-like plasmids encoding blaCTX-M-65 [7]. Masoud et al. identified extended-spectrum beta-lactamases (ESBLs), metallo beta-lactamases (MBLs), and plasmid-mediated quinolone resistance in *E. coli* isolated from different clinical specimens in Egypt [14].

*Klebsiella pneumoniae* is another important human pathogen and one of the etiological agents of pneumonia. Mendes et al. identified Ceftazidime/Avibactam resistance in a patient isolate in Portugal [4]. As the combination resistance to Ceftazidime and Avibactam is new, and as it appears together with resistance to other antibiotics, the authors recommend special epidemiological surveillance.

Altayb et al. studied and isolated a *K. pneumoniae* isolate from a patient with recurrent urinary tract infection, identified as hypermucoviscent, type 2 (K2) capsular polysaccharide, ST14, and multidrug-resistant. The authors performed genomic analysis and demonstrated that it harbors four plasmids, several virulence factors, and antibiotic resistance encoding genes, which are worth studying in more detail [5].
